# Allelic overload and its clinical modifier effect in Bardet-Biedl syndrome

**DOI:** 10.1038/s41525-022-00311-2

**Published:** 2022-07-14

**Authors:** Irene Perea-Romero, Carlos Solarat, Fiona Blanco-Kelly, Iker Sanchez-Navarro, Brais Bea-Mascato, Eduardo Martin-Salazar, Isabel Lorda-Sanchez, Saoud Tahsin Swafiri, Almudena Avila-Fernandez, Inmaculada Martin-Merida, Maria Jose Trujillo-Tiebas, Ester Carreño, Belen Jimenez-Rolando, Blanca Garcia-Sandoval, Pablo Minguez, Marta Corton, Diana Valverde, Carmen Ayuso

**Affiliations:** 1 Department of Genetics, Health Research Institute-Fundación Jiménez Díaz University Hospital, Universidad Autónoma de Madrid (IIS-FJD, UAM), Madrid, Spain; 2 Center for Biomedical Network Research on Rare Diseases (CIBERER), Instituto de Salud Carlos III, Madrid, Spain; 3 CINBIO, Universidade de Vigo, Vigo, Spain; 4 Instituto de Investigación Sanitaria Galicia Sur (IIS Galicia Sur), Álvaro Cunqueiro Hospital, Vigo, Spain; 5 Department of Ophthalmology, Fundación Jiménez Díaz University Hospital (FJD), Madrid, Spain

**Keywords:** Molecular medicine, Hereditary eye disease, Personalized medicine

## Abstract

Bardet–Biedl syndrome (BBS) is an autosomal recessive ciliopathy characterized by extensive inter- and intra-familial variability, in which oligogenic interactions have been also reported. Our main goal is to elucidate the role of mutational load in the clinical variability of BBS. A cohort of 99 patients from 77 different families with biallelic pathogenic variants in a BBS-associated gene was retrospectively recruited. Human Phenotype Ontology terms were used in the annotation of clinical symptoms. The mutational load in 39 BBS-related genes was studied in index cases using different molecular and next-generation sequencing (NGS) approaches. Candidate allele combinations were analysed using the in silico tools ORVAL and DiGePred. After clinical annotation, 76 out of the 99 cases a priori fulfilled established criteria for diagnosis of BBS or BBS-like. *BBS1* alleles, found in 42% of families, were the most represented in our cohort. An increased mutational load was excluded in 41% of the index cases (22/54). Oligogenic inheritance was suspected in 52% of the screened families (23/45), being 40 tested by means of NGS data and 5 only by traditional methods. Together, ORVAL and DiGePred platforms predicted an oligogenic effect in 44% of the triallelic families (10/23). Intrafamilial variable severity could be clinically confirmed in six of the families. Our findings show that the presence of more than two alleles in BBS-associated genes correlated in six families with a more severe phenotype and associated with specific findings, highlighting the role of the mutational load in the management of BBS cases.

## Introduction

Bardet–Biedl syndrome (BBS, MIM #209900) is a rare multisystemic disease that is caused by the dysfunction of primary cilia^1^. BBS is a complex ciliopathy mainly characterized by progressive retinal dystrophy, postaxial polydactyly, obesity, hypogonadism, renal anomalies, and cognitive impairment^1^. Additional findings are type 2 diabetes mellitus, speech or developmental alterations, dental anomalies, brachydactyly/syndactyly, ataxia, anosmia/hyposmia, heart malformations, or Hirschsprung disease^1,2^. Its incidence varies from 1:160,000 in northern Europe^3^ to 1:13,500–18,000 in several isolated communities with higher rates of inbreeding^4,5^.

Genetically, BBS is also a heterogeneous disorder with 24 loci associated to date, according to the data extracted from OMIM (Online Mendelian Inheritance in Man; last accessed February 2022) and the Human Gene Mutation Database Professional (HGMD) 2021.4 database (last accessed in February 2022). The first 21 loci (BBS1-21) account for ~80% of all the cases diagnosed with this syndrome^6^. BBS-related genes encode for proteins of the primary cilium and the basal body complex^7^. Besides systemic forms, some BBS genes have also been linked to non-syndromic retinopathies, as well as other systemic ciliopathies, such as Joubert (JBTS, MIM #213300), McKusick-Kaufman (MKKS, MIM #236700), Meckel (MKS, MIM #2490000), and Senior-Løken (SLSN, MIM #266900) syndromes.

BBS is usually inherited as an autosomal recessive Mendelian trait with variable intra- and inter-familial severity^2^. Twenty years ago, triallelism, i.e., three alleles in two BBS loci, was first described in a BBS family^8^. An unaffected sibling carried two variants in *BBS2* (MIM *606151), whereas the BBS-affected patient additionally presented the third allele in *MKKS* (MIM *604896)^8^. However, subsequent studies have found no evidence of its existence^9,10^. Regardless of triallelism, in other cases, the penetrance and/or phenotypic expressivity of causative biallelic BBS variants are modulated in some families by the presence of oligogenic modifiers^11–14^. In those families, a third mutation in a second gene has been correlated with an earlier onset or a more severe specific phenotype in the carrier BBS patients due to a probable modifying effect^13–15^.

Variants in the secondary gene are not always straightforwardly predicted to be pathogenic since they can also be hypomorphic or common alleles with high population frequencies that are a priori considered benign. To characterize the interaction, in vitro^16^ and in vivo^17^ models are normally used to assess allelic combinations and their effect on modulating clinical outcomes. As functional studies are not always feasible in a clinical setting, emerging in silico tools, such as ORVAL (Oligogenic Resource for Variants Analysis)^18^ or DiGePred (DiGenic Predictor)^19^, could be useful to help predict the effect of the mutational burden of rare and common variants.

This work focuses on establishing and understanding new potential oligogenic combinations that may explain the clinical variability in BBS-affected families. The identification of new possible modifier alleles may also have an important impact on genetic counseling and clinical management.

## Results

### Clinical description of the cohort and genetic outcome

**Fig. 1:**
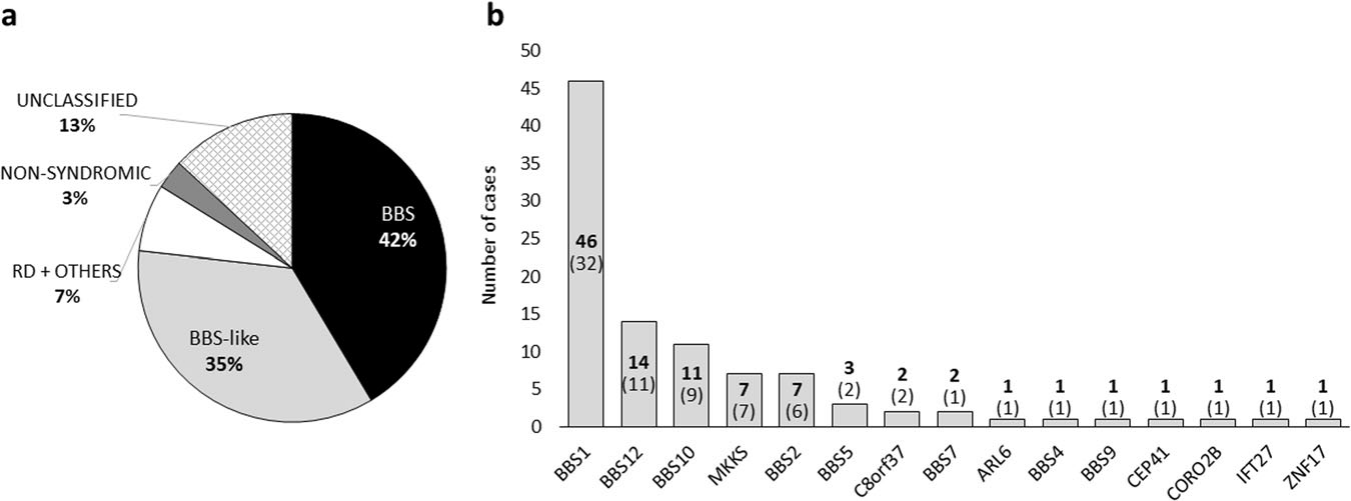
Clinical classification and genetic distribution of the cohort. **a** A priori clinical classification. All patients have been classified into five categories: (i) Bardet–Biedl syndrome (“BBS”); (ii) suspected of BBS (“BBS-like”); (iii) “RD + OTHERS”; (iv) “NON-SYNDROMIC”; and (v) “UNCLASSIFIED”. **b** Distribution of the causative biallelic genes found in the complete cohort. Bold numbers indicate the total number of cases genetically diagnosed for each gene, while the number of families to which these cases belong is indicated in brackets.

This study retrospectively included 99 affected individuals (77 probands and 22 affected siblings) from 77 families with a final genetic diagnosis of BBS. The cohort consisted of 54 males and 45 females (mean age: 42.2 ± 16.1 years old). Patients were phenotypically classified considering HPO terms and specific clinical criteria for BBS/BBS-like (Supplementary Table 1). After clinical annotation, the most frequent features in the whole cohort were RD, obesity, and postaxial polydactyly, which appeared in more than 80% of the syndromic cases with available clinical information (*n* = 83) (Supplementary Table 3). 77% (76/99) of the cases in our cohort fulfilled a priori the diagnosis criteria for BBS or BBS-like (Fig. 1a). Among the patients who did not meet the minimum criteria for BBS/BBS-like diagnosis prior to the final molecular diagnosis, seven cases presented visual alteration together with a combination of extra-ocular features not specific for BBS, and they were therefore classified in the group “RD + OTHERS”. Besides, three affected siblings suffered from isolated RD and then, were classified as “NON-SYNDROMIC”. Finally, no clinical data were available for 13 patients, so they were not clinically classified.

After molecular testing, a total of 15 biallelic disease-causing genes were found. The most mutated gene was *BBS1*, appearing in 42% of the families (*n* = 32), followed by *BBS12* (14%) and *BBS10* (12%). Eight genes were found in only one family (Fig. 1b). A total of 57 different alleles were found in these 15 genes, being the missense variant p.(Met390Arg) in *BBS1* the most frequently identified among the causative alleles (32%; 64/198). The 6 more frequently identified alleles accounted for half of all alleles found (100/198) (Supplementary Table 4).

### Oligogenic outcome

**Fig. 2:**
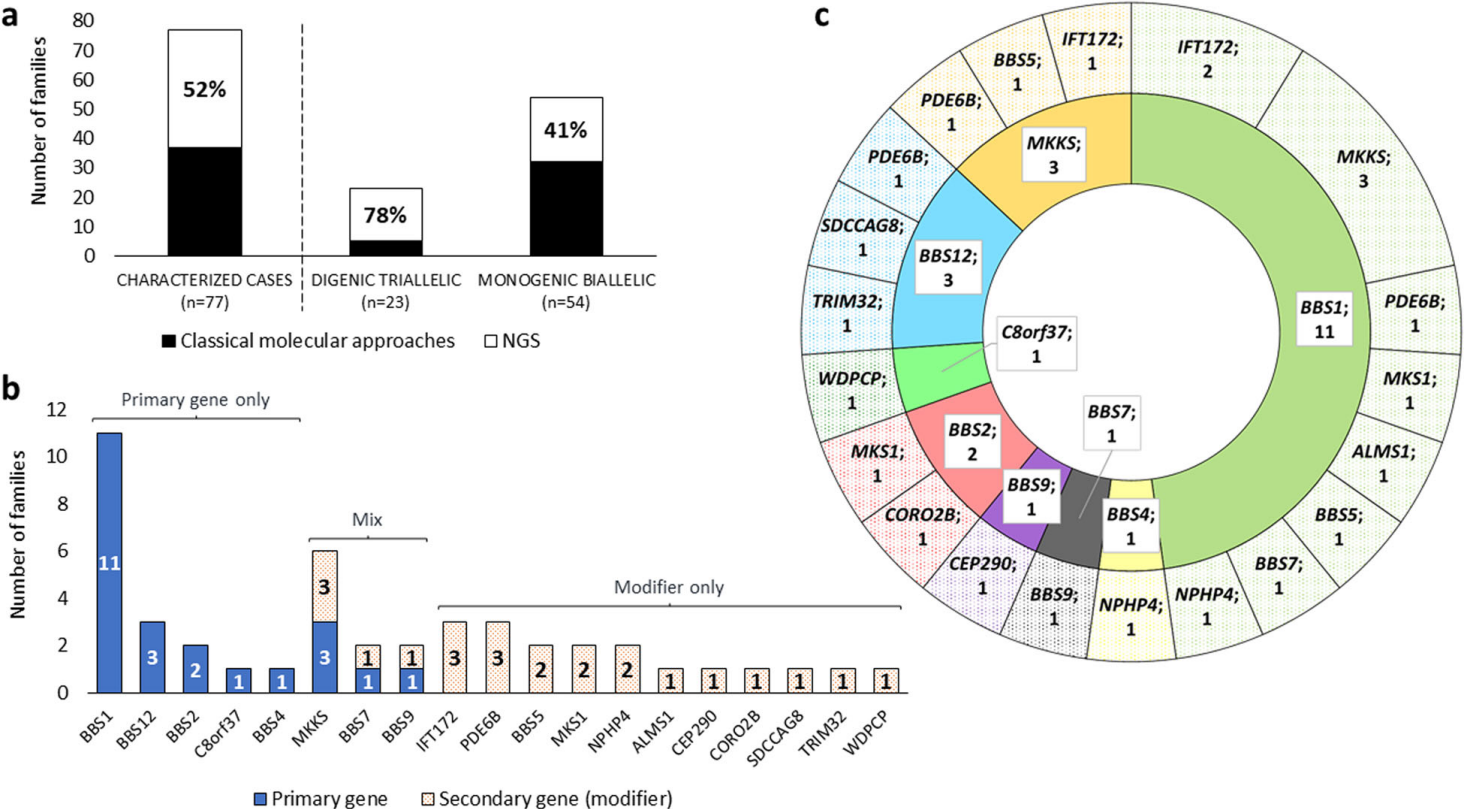
Mutational load in BBS-related genes. **a** Distribution of BBS families according to the molecular screening and mutational load. Families are grouped as “digenic triallelic” (three mutant alleles in two recessive genes) and “monogenic biallelic” (two mutant alleles in a recessive gene). NGS includes customized targeted NGS panel, clinical exome sequencing (CES), and/or whole-exome sequencing (WES). **b** Mutational load and gene role in the allelic combination. The genes found in the “digenic triallelic” families are grouped considering if they are primary (recessive biallelic gene) or secondary (monoallelic modifier) genes. **c** Distribution of the oligogenic cases according to the primary and secondary genes found in the 23 families with suspected triallelic inheritance. Only the genetic outcome of each proband is shown. The inner and outer circles represent the biallelic primary gene and the monoallelic gene (or possible modifier) accompanying the biallelic variants, respectively.

In our cohort of families carrying biallelic variants in a primary BBS gene, we assessed the existence of third alleles in other BBS genes to be candidates as genetic modulators. In 40 probands with available NGS data from almost all BBS genes, an NGS reanalysis was performed to explore their mutational load in BBS-related genes. For the remaining 37 index cases, the search of third alleles has been restricted to those BBS genes screened by genotyping microarrays and Sanger sequencing (Fig. 2a). Families were grouped according to their mutational load after all the genetic studies in “digenic triallelic” (*n* = 23) or “monogenic biallelic” (*n* = 54) families. A total of 21 different potential monoallelic modifiers were found in 14 genes. The missense variant p.(Ala242Ser) in *MKKS* was the most prevalent variant among modifier alleles (16%; 4/25) (Supplementary Table 4).

NGS data allowed to screen a larger number of genes than traditional methods. As a result, in the 40 cases studied by NGS, there was a higher proportion of families falling into the “digenic triallelic” subgroup (78%) than into the “monogenic biallelic” subgroup (41%) (Fig. 2a).

The overall oligogenic rate was 51% (23/45). Indeed, after NGS reanalysis, 18/40 families were positive for the third allele in a secondary BBS-related gene, plus five additional oligogenic positive families that were identified by traditional methods (5/37). The presence of a potential modifier allele could only be excluded in 41% (22/54) of the cases, which had been analysed by NGS as a first-tier approach or reanalyzed to assess the presence of additional alleles in already known BBS-related genes. Nevertheless, the 32 biallelic probands only screened by traditional methods could not be excluded from participating in a potential triallelic inheritance.

Regarding mutational load, *BBS1*, *BBS12*, *BBS2*, *C8orf37* (MIM *614477), and *BBS4* (MIM *600374) always appeared as the main cause of disease, e.g., primary gene, in the “digenic triallelic” cases (Fig. 2b), whereas *MKKS*, *BBS7* (MIM *607590), and *BBS9* (MIM *607968) could take all allelic roles. Besides, eleven genes have only been found as possible modifiers.

**Fig. 3:**
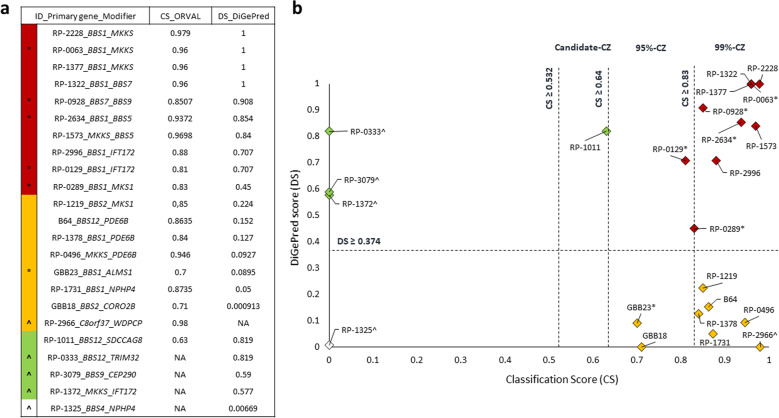
Predicted oligogenic effects of allelic combinations in BBS-related genes. **a** List of the allelic combination of the 23 families and their DiGePred and ORVAL scores. The families were grouped according to the scores obtained in those two in silico tools in (i) high confidence in both methods (red color), gene pair with digenic disease potential (DiGePred Score (DS) ≥0.374), and predicted oligogenic combination of variants with a confidence of at least 95% (Classification Score (CS) ≥0.64); (ii) high confidence for genes with digenic potential, DS ≥0.374 (green); (iii) high confidence for the oligogenic combination, but in genes with lower potential for digenism, 95%-zone candidate in ORVAL (CS ≥0.64) (orange); and iv) combinations in which digenic potential is discarded (white). ^Predictions in one of the methods could not be made for five allelic combinations. *Six families in which phenotypic and allelic differences between affected siblings were reported. **b** Graphic representation of the scores obtained in DiGePred and ORVAL.

Twenty different combinations of two BBS genes were identified within the triallelic families, being *BBS1* the most frequently involved in 11 of them as the primary gene. Three genes were overrepresented as modifiers compared to the rest, which were *MKKS*, *IFT172* (MIM *607386), and *PDE6B* (MIM *180072). In addition, only the combination of *BBS1*_*MKKS* (*n* = 3) and *BBS1*_*IFT172* (*n* = 2) appeared more than once (Fig. 2c). However, each allelic combination was private, so no common distribution of alleles between families was found (Supplementary Tables 5, 6). All these gene pairs and allelic combinations were rated using the DiGePred classifier and ORVAL platform, respectively. However, predictions could not be made for one of the 23 gene pairs and for four of the 23 allelic combinations (Fig. 3a). According to the potential oligogenic pathogenic effects, 44% (10/23) of the identified triallelic combinations were predicted to have an oligogenic inheritance with both methods with a 95% of confidence in ORVAL and the highest confidence threshold in DiGePred. Besides, the possibility of oligogenic inheritance reached 91% at the same confidence when only one method was considered (Fig. 3a, b).

There were no significant differences in the distribution of symptoms between the cases with respect to their detected mutational load. However, there was slight enrichment in the frequency of aganglionic megacolon in “digenic triallelic” cases, and brachydactyly in “monogenic biallelic” (Supplementary Table 3).

### Evidence of modifier alleles in our cohort

Among the 23 families in which a third allele apart from biallelic disease-causing variants was identified, we assessed the possibility of being true oligogenic modifiers in view of several facts, such as the oligogenic effect predicted by ORVAL and DiGePred (Fig. 3), the informativity of the family, and intrafamilial differences in the penetrance of major BBS features and/or the severity within the affected individuals.

We found three families with differences in the severity of their syndromic-associated symptoms (family IDs GBB23, RP-0063, and RP-0928), having the triallelic index case a more severe presentation than its biallelic sibling (Supplementary Table 5). First, in the family GBB23 with two siblings with biallelic *BBS1* variants causing a clinical diagnosis of BBS, the proband also carries a third missense allele in *ALMS1* (p.(His3880Tyr)), developed type 2 diabetes mellitus (T2DM). Only ORVAL predicted the *BBS1_ALMS1* combination as oligogenic with a 95% of confidence (CS = 0.7). Secondly, biallelic *BBS1* variants were found in the two siblings affected by BBS of family RP-0063. The proband, which also presented polydactyly, intellectual disability, renal anomalies, asthma, and seizures, carried the third allele in *MKKS* (p.(Ala242Ser)). This combination was predicted to be oligogenic by ORVAL with 99% of confidence (CS = 0.96) and DiGePred (DS = 1). Finally, family RP-0928 was formed by two BBS-affected siblings with biallelic *BBS7* variants. In the proband, who also exhibited hepatic steatosis, an additional allele in *BBS9* (p.(Met126Leu)) was found. This combination had a CS and a DS of 0.8507 and 0.908, respectively. It was therefore predicted as oligogenic with 99% of confidence by the ORVAL platform.

Interestingly, an oligogenic effect was also suspected in 3 other families (family IDs RP-0129, RP-0289, and RP-2634) due to the absence of syndromic features in one biallelic affected sibling, who only suffered from isolated RD, while the triallelic proband had syndromic clinical features (Supplementary Table 5). ORVAL with 95% confidence and DiGePred predicted a possible oligogenic effect in all combinations with possible triallelic inheritance in the probands of these families (Supplementary Table 5 and Fig. 3).

The modifier effect in 17 out of 23 suspected triallelic inheritance remained unclear due to the lack of familial informativeness and/or clinical data. While individuals from the families RP-1322, RP-1377, and RP-1378, both presented the same genotype and/or syndromic phenotype (Supplementary Table 5), triallelic inheritance was found in 13 sporadic cases with no other affected relative. Furthermore, there was a lack of clinical information in an additional family (B64). However, ORVAL (95%-candidate zone) together with DiGePred predicted a likely oligogenic effect in five allelic combinations found in these families, and only one of those platforms in 11 more (Fig. 3).

## Discussion

BBS is an extremely clinically and genetically heterogeneous ciliopathy characterized by intra- and inter-familial variability^2,20^. Generally, BBS presents with an autosomal recessive inheritance, but in some cases, an oligogenic inheritance has been proposed, in the form of triallelism or second-site modifiers^8,11–13,21^. In these families, some unaffected or less severely affected individuals have two pathogenic variants in a BBS-related gene, whereas the BBS-diagnosed or more severely affected relatives carry three alleles in two different BBS-related genes. Although the involvement of triallelism in BBS families is controversial^8–10,13^, there is further evidence for the possible existence of third modifier alleles^11–14^. Hence, our study presents a comprehensive study of the mutational burden in BBS and highlights the importance of considering non-Mendelian inheritance to improve the clinical management of BBS.

First, we recruited a large cohort of 99 cases from 77 families with genetic suspicion of BBS after molecular testing. Although other studies only consider those cases that met the diagnostic criteria for BBS^10,12,13^, we have included also patients who did not fulfill a priori the clinical criteria of BBS described by ref*.* ^1^. In fact, half of the syndromic cases with available clinical information (41/83) were not first classified as BBS but after genetic testing. Thus, for some of the clinical features, a slight bias was observed in our cohort compared to the distribution in other reports^1,2,22^, which can be explained by the inclusion of cases with a diagnosis of “BBS-like” or even more unspecific systemic findings (“RD-OTHERS”), or a poor clinical acquisition of some of the features.

Nowadays, NGS is the technology of choice for the study of BBS^23–25^. It allows the identification of new causative variants and further reanalysis to assess new BBS genes that may have been identified after a primary analysis or had not been covered by any of the classical methods initially used^26,27^. This statement is consistent with the fact that 78% of the suspected oligogenic families in our cohort were discovered through NGS approaches.

It has been estimated that oligogenic inheritance is present in less than 10% of the BBS families^28^. This value is confirmed by our triallelic distribution, with a rate of 13% in the informative triallelic families. However, it increases to 51% when all families with suspected modifiers alleles were included. We cannot elucidate if the third allele triggers a modifier effect in most families, because both siblings presented the same genotype-phenotype, the index case was the only affected in the family, or clinical information was unavailable.

*BBS1* can cause both BBS and non-syndromic inherited RD. This gene is the most frequent source of BBS, accounting for 23–51% of characterized families^12,29,30^, which agrees with 42% of molecularly characterized families with *BBS1* as the major primary gene in our cohort. The variant p.(Met390Arg) has been reported in up to 80% of *BBS1*-related alleles across different worldwide populations^29^, being mostly associated with BBS as only 21% of *BBS1*-positive patients showed non-syndromic presentations^31^. In our cohort, this variant is accordingly the most represented with an allelic frequency of 70%. The reason for this slight decrease may be that we only included BBS-associated families but excluded those with only non-syndromic affected individuals.

The implication of *BBS1* in oligogenic inheritance remains unclear. Some reports claim that *BBS1* is rarely involved in complex inheritance^29^, but *BBS1* has been reported in a triallelic inheritance in 15% of families, being the primary gene instead of acting as a modifier in only 4% of cases^12^. However, our results go further, showing that only 65% of our *BBS1*-characterized families fit in an autosomal recessive inheritance, while in the remaining families, *BBS1* might participate in oligogenic inheritance as the primary gene in 11 families, together with *MKKS*, *IFT172*, or other six BBS-related genes. This same behavior was observed for *BBS12* in three families from our cohort, but in this case, there are no reports related to the likelihood of its participation in complex inheritance. In those families, biallelic *BBS12* variants were found together with the missense variants p.(Gly352Val) in *PDE6B*, p.(Arg82Leu) in *TRIM32* (MIM *602290), and p.(Arg400Cys) in *SDCCAG8* (MIM *613524), respectively. While the combinations *BBS12_SDCCAG8* and *BBS12_TRIM32* were predicted only with DiGePred, only the combination *BBS12_PDE6B* was predicted as oligogenic with ORVAL platform with a 99% of confidence.

Generally, BBS-associated proteins are located at the base of the cilium and participate in ciliary biogenesis and in cilia function^1,32^, but a variety of specific locations and functions have been described^33,34^. In our work, the involvement of BBS proteins in a specific complex, structure, or process does not seem to be related to the level of involvement of each gene in the triallelic inheritance. However, we have seen that the genes that encode for the chaperonin-like complex (*BBS10*, *BBS12*, and *MKKS*) are mostly implied in a recessive inheritance. Moreover, *BBS12* and *MKKS* are normally the primary genes when involved in oligogenic inheritance. Therefore, these genes are usually the principal gene causing the disease regardless of the type of inheritance. It has been reported that the activity of genes encoding BBSome components (*ARL6, BBIP1, BBS1, BBS2, BBS5, BBS7, BBS9*, and *TTC8*) may be dependent on the chaperonin-like genes^35^ and families with variants in the chaperonin-like complex present a more severe phenotype^36^, so these chaperonin-like genes may not normally require a second-site modifier. Nevertheless, we found three families from our cohort carrying the variant p.(Ala242Ser) in *MKKS* as a possible modifier allele in combination with biallelic *BBS1* variants. This non-synonymous change, which has been previously described as a dominant-negative allele^17,37^, disrupts the protein conformation of the BBSome, thus preventing them from doing their proper function^17,37^. Intrafamilial variable severity has been seen in one of these three families carrying the p.(Ala242Ser) variant in combination with homozygous p.(Met390Arg) in *BBS1*. Our result suggests that the increase in the detected mutational load may correlate with a more severe phenotype, which could be explained by its chaperone function^38^. Variable expressivity between siblings involving other genetic combinations can be also found in the other five families in our cohort.

The presence of modifier alleles can determine the phenotype, since they may influence the presentation of the BBS phenotype^17^. This can be the scenario for family RP-2634, in which the syndromic index case has biallelic variants in *BBS1* and the heterozygous missense p.(Arg207His) in *BBS5*, whereas her non-syndromic sister is just biallelic for the *BBS1* variants. This *BBS5* variant, presenting a minor-allele frequency of 0.9% in Europeans, has been predicted as a null mutation^17^.

Specific heterozygous variants acting as modifiers has previously been associated with the existence of particular findings (e.g., ocular, neurological, or renal features)^39–41^. A sibling from family GBB23 carries the homozygous pathogenic variant p.(Met390Arg) in *BBS1*, and additionally, the index case suffering from T2DM also carries the heterozygous variant p.(His3880Tyr) in *ALMS1*. Mutations in *ALMS1* are the cause of Alström syndrome (ALMS; MIM #203800), an ultra-rare metabolic ciliopathy associated with severe visual impairment, sensorineural deafness, obesity, insulin resistance, T2DM, and hypogonadism, among other features^42^. One of the explanations of glucose metabolism alterations in this syndrome are defects in the ALMS1 protein, which participates in the insulin-regulated glucose transport^43^. In our family, the variant identified in *ALMS1* may be acting as a second-site modifier altering the possibility of suffering T2DM. However, the high frequency of diabetes mellitus in the general population^44^ might also be a plausible explanation for its presence in this case.

Some of the possible modifier alleles in the BBS-causing genes found in our cohort could be good candidates for functional studies to analyse a possible modifying effect on the BBS phenotype, e.g., *IFT172*, *TRIM32*, or *WDPCP*. Furthermore, we identified third alleles in other genes previously reported as possible candidates or modifiers of BBS, e.g., *ALMS1*, *CORO2B*, *NPHP4*, or *PDE6B*^36,45–47^. Therefore, our findings could also support a possible involvement of these genes in the pathogenesis of BBS. For example, *PDE6B* is a gene associated with non-syndromic RD but also reported to BBS phenotype in a consanguineous family with homozygous pathogenic mutations in *BBS10* and *PDE6B*^47^. Here, three different heterozygous variants in *PDE6B* were found in three families (family IDs RP-1378, RP-0496, and B64), accompanying biallelic variants in *BBS1*, *MKKS*, and *BBS12*, respectively. It is unknown how the effect of the mutational load detected in these families may be affecting their phenotype, but *PDE6B* and other genes, which are involved in the phototransduction and visual transduction pathways, are downregulated in BBS and ALMS zebrafish models and may be drivers of the retinal degeneration^48^. Nonetheless, the hypothetical role of *PDE6B* and other unclear modifiers in BBS should be further studied functionally.

The effect of modifier alleles on clinical manifestation needs to be assessed usually by means of in vitro and in vivo strategies^16,17^. However, due to technical limitations, it is not always possible to perform these analyses in a clinical setting. Alternatively, in silico tools can help to discover and predict combinations of variants that can be affecting the patients’ phenotype^18,19^. According to our data, ORVAL and DiGePred together support an oligogenic inheritance in 44% of our families with more than two alleles, reaching 91% with only one positive method.

To understand the phenotypic variability in BBS families, both genetic and environmental factors should be considered. Recently, multi-omics analyses are being considered to understand and elucidate the role of the mutational load in BBS-associated mechanisms through the integration of multiple analyses (mutational load, differential gene and/or protein expression, epigenetic, and/or metabolome-based signatures). Therefore, obtaining larger data sets would help to clarify the role of possible modifiers in BBS and related ciliopathies^23,49^.

In summary, this work deepens into the controversial topic of oligogenic inheritance in BBS, finding new evidence for the existence of second-site genetic modifiers as a cause of intrafamilial variability in this disease. Besides, it highlights the importance of the use of NGS in the genetic diagnosis of BBS.

## Methods

### Subjects and phenotypic classification

This research has been reviewed and approved by the Research Ethics Committee of the Fundación Jiménez Díaz University Hospital (FJD, Madrid, Spain) (approval number PIC172-20_FJD) and the Galician Ethical Committee for Clinical Research (Spain-no. 2006/08) following the principles of the Declaration of Helsinki and its further revisions. Written informed consent was collected from all patients, or their legal guardians, when necessary, prior to inclusion in the study.

Families were retrospectively recruited from patient registries at FJD and through collaborators from different Spanish hospitals and research institutions over the last 30 years^31^. The inclusion criteria for families was a genetic diagnosis of BBS in at least one affected member. Available clinical and familial data were extracted for each patient and reviewed through medical reports, questionaries, and/or electronic health records, as previously described^38,50^.

Clinical data from all affected individuals were annotated using Human Phenotype Ontology (HPO) terms. All cases, including probands (cases with the more severe phenotype) and their affected relatives, were then classified into five different phenotypic subgroups: (i) “BBS” and (ii) “BBS-like” when the patient fulfilled the clinical diagnostic criteria previously specified in ref. ^51^. (Supplementary Table 1); (iii) “Retinal dystrophy (RD) + OTHERS”, when a syndromic patient did not meet the BBS/BBS-like minimum criteria; (iv) “NON-SYNDROMIC”, when relatives of a BBS/BBS-like/”RD + OTHERS” index case suffered from RD without extra-ocular features; and (v) “UNCLASSIFIED”, which contained patients with no clinical data available.

### Molecular analysis

Diagnostic genetic testing was performed in probands using different molecular approaches over the years. These included commercial genotyping microarray for known pathogenic variants in 12 BBS genes (Asper Biotech, Estonia), Sanger sequencing of the mayor BBS genes (*BBS1* (MIM *209901), *BBS10* (MIM *610148), and *BBS12* (MIM *610683)), and/or NGS approaches, such as customized targeted NGS panels, clinical exome sequencing (CES), and/or whole-exome sequencing (WES), as previously reported^36,38,51–55^.

### Oligogenic analysis

The available NGS data from probands were analysed to explore the mutational load in BBS-related genes. We looked for possible third alleles in other BBS genes, which could act as modulators of the recessive biallelic variants at the primary gene. In the 52% of probands (40/77) with available NGS data, an NGS reanalysis was performed using specific subpanels of 29 and 37 genes for prioritizing rare variants in CES and WES analysis, respectively (Supplementary Table 2). These virtual panels were composed of already known disease-causing genes or known modifiers of BBS/BBS-like phenotypes after a literature revision, as well as retrieved from OMIM, HGMD, and RetNet databases (The Retinal Information Network, https://sph.uth.edu/retnet/; last accessed February 2022).

A monoallelic variant was considered a candidate for being a modifier allele when it was classified as class 3, 4, or 5 according to the recommendations of the American College of Medical Genetics and Genomics (ACMG)^56^, or when it had been previously hypothesized as a modifier allele in the literature. All these putative modifier alleles and the primary biallelic disease-causing variants were confirmed and segregated by Sanger sequencing in the available affected and unaffected family members.

After genetic analysis, families were classified according to their detected mutational load in “digenic triallelic” if three mutant alleles were found in two recessive genes, or “monogenic biallelic” if only two mutant alleles were found in a recessive BBS gene.

To consider a family as possibly triallelic, it had to have at least two affected relatives with differences in their genotype (e.g., proband triallelic with a biallelic sibling) and in their corresponding phenotype.

The involved genes and the three mutant alleles of each “digenic triallelic” case were analysed using two different in silico tools, to predict candidate gene pairs and pathogenic allele combinations that could be participating in the intrafamilial variability between BBS probands and their affected siblings. All gene pairs of probands from families with suspected oligogenic inheritance were assessed using the machine learning tool DiGePred (http://www.meilerlab.org/index.php/servers; last accessed March 2022)^19^, following its “random” model, which classified gene pairs as digenic when the DiGePred value was equal or greater than 0.374. Additionally, all allelic combinations of probands were analysed in silico using the ORVAL platform (https://orval.ibsquare.be/; last accessed March 2022)^18^. Since all combinations shared a Support Score of 100% in the ORVAL tool, the Classification Score (CS) was used as the sole predictor of the probability that each specific combination was disease-causing: (i) confidence of 99% of being a candidate (CS ≥0.83); (ii) 95%-zone candidate (CS ≥0.64); and (iii) candidate (CS ≥0.532).

### Statistical analysis

To determine the changes in the number of symptoms of the different subgroups of patients according to the genetic outcome, a chi-square test was carried out and *p* values under 0.05 were considered statistically significant.

### Reporting summary

Further information on research design is available in the Nature Research Reporting Summary linked to this article.

## Supplementary information

Supplementary Information

Supplementary Table S4

Supplementary Table S5

Supplementary Table S6

Reporting Summary Checklist

## Data Availability

NGS data were available in public, open access repositories such as the European Genome-Phenome Archive (EGA; https://www.ebi.ac.uk/ega/home; EGAD00001007022 and EGAS00001006368) and the Collaborative Spanish Variant Server (CSVS; http://csvs.babelomics.org/) as aggregated data. The rest of the data were available upon reasonable request.

## Code availability

The pipeline used for WES data analysis (VariantCallingFJD-v2.0.0) is available online at https://github.com/TBLabFJD/VariantCallingFJD.
