# Supplementary material for: Allelic overload and its clinical modifier effect in Bardet-Biedl syndrome
Source: NPJ Genom Med. 2022 Jul 14;7:41. doi: 10.1038/s41525-022-00311-2 (PMC9283419; doi:10.1038/s41525-022-00311-2)
Supplement: Supplementary file 1 — Supplementary Information [file 41525_2022_311_MOESM1_ESM.pdf]

Allelic overload and its clinical modifier effect in Bardet-Biedl syndrome

Perea-Romero I[1,2], Solarat C[3,4], Blanco-Kelly F[1,2], Sanchez-Navarro I[1], Bea-Mascato B[3,4], Martin-Salazar E[3,4], Lorda-Sanchez I[1,2], Tahsin-Swafiri S[1,2], Avila-Fernandez A[1,2], Martin-Merida I[1,2], Trujillo-Tiebas MJ[1,2], Carreño E[5], Jimenez-Rolando B[5], Garcia-Sandoval B[2,5], Minguez P[1,2], Corton M[1,2], Valverde D[3,4,*], Ayuso C[1,2,*]

[1] Department of Genetics, Health Research Institute-Fundación Jiménez Díaz University Hospital, Universidad Autónoma de Madrid (IIS-FJD, UAM), Madrid, Spain

[2] Center for Biomedical Network Research on Rare Diseases (CIBERER), Instituto de Salud Carlos III, Madrid, Spain

[3] CINBIO, Universidade de Vigo, Vigo, Spain

[4] Instituto de Investigación Sanitaria Galicia Sur (IIS Galicia Sur), Álvaro Cunqueiro Hospital, Vigo, Spain

[5] Department of Ophthalmology, Fundación Jiménez Díaz University Hospital (FJD), Madrid, Spain

* Corresponding authors. E-mails: dianaval@uvigo.es (DV) and cayuso@fjd.es (CA)

Supplementary Table 1. Clinical criteria for the diagnosis of Bardet-Biedl syndrome (BBS) or BBS-like [1]

	Major finding (MC)	Minor finding (mc)	Supportive evidence (SE)	Disease diagnostic criteria	Disease-like diagnostic criteria
BBS [2]	- Rod-cone dystrophy<br>- Postaxial polydactyly<br>- Obesity<br>- Abnormality of the female and male genitalia<br>- Abnormality of the kidney<br>- Specific learning disability, intellectual disability	- Delayed speech and language development<br>- Global developmental delay<br>- Type II diabetes mellitus<br>- Abnormality of the teeth<br>- Abnormal heart morphology<br>- Brachydactyly syndrome, syndactyly<br>- Ataxia, poor coordination<br>- Anosmia, hyposmia	- Chronic otitis media<br>- Aganglionic megacolon (Hirschprung disease)	a) 4 MC<br>or<br>b) 3 MC and 2 mc	a) At least, 3 MC and 1 mc<br>or<br>b) less MC, but including postaxial polydactyly

1. Perea-Romero, I. *et al.* NGS and phenotypic ontology-based approaches increase the diagnostic yield in syndromic retinal diseases. *Hum Genet* **140**, 1665–1678 (2021).
2. Forsythe, E. & Beales, P. L. Bardet-Biedl syndrome. *Eur J Hum Genet* **21**, 8–13 (2013).

Supplementary Table 2. List of the genes included in the 29-gene subpanel for clinical exome sequencing (CES) and 37-gene subpanel for whole exome sequencing (WES). Already reported disease-causing genes and modifiers of BBS have been included. From the 40 out of 77 families that were studied using NGS, 33 had only CES and the remaining 7 had WES

	Disease-causing gene	Modifier	CES	WES
ADIPOR1 (NM_015999.6)	X		X	X
ALMS1 (NM_015120.4)	X	X	X	X
ARL6 (NM_177976.3)	X	X	X	X
BBIP1 (NM_001195306.1)	X			X
BBS1 (NM_024649.5)	X	X	X	X
BBS10 (NM_024685.4)	X		X	X
BBS12 (NM_152618.3)	X		X	X
BBS2 (NM_031885.5)	X		X	X
BBS4 (NM_033028.5)	X	X	X	X
BBS5 (NM_152384.3)	X	X	X	X
BBS7 (NM_176824.3)	X	X	X	X
BBS9 (NM_198428.3)	X	X	X	X
C8orf37 (NM_177965.3)	X	X	X	X
CCDC28B (NM_024296.5)		X	X	X
CEP164 (NM_014956.5)	X			X
CEP19 (NM_032898.5)	X			X
CEP290 (NM_025114.4)	X	X	X	X
CEP41 (NM_018718.3)	X		X	X
CORO2B (NM_006091.5)	X			X
IFT172 (NM_015662.3)	X		X	X
IFT27 (NM_006860.5)	X			X
IFT74 (NM_025103.4)	X			X
KIF7 (NM_198525.3)	X		X	X
LZTFL1 (NM_020347.4)	X		X	X
MKKS (NM_170784.3)	X	X	X	X
MKS1 (NM_017777.4)	X		X	X
NPHP1 (NM_001128178.3)	X		X	X
NPHP4 (NM_015102.5)	X	X	X	X
PDE6B (NM_000283.4)	X		X	X
SCAPER (NM_020843.4)	X			X
SCLT1 (NM_144643.4)	X			X
SDCCAG8 (NM_006642.5)	X	X	X	X
TMEM67 (NM_153704.6)	X	X	X	X
TRIM32 (NM_001099679.2)	X		X	X
TTC21B (NM_024753.5)	X		X	X
TTC8 (NM_144596.4)	X		X	X
WDPCP (NM_015910.7)	X		X	X

Supplementary Table 3. **Summary of the clinical data of the 83 patients presenting systemic features.** Three non-syndromic cases of retinal dystrophies and 13 unclassified cases due to the lack of clinical information were not included. After oligogenic analyses of BBS-related gene, cases were classified according to their detected mutational load in: i) "digenic triallelic (n = 24) or ii) "monogenic biallelic" (n = 21). In the remaining 38 cases, no oligogenic studies were performed. Clinical presentation of all patients was annotated using Human Phenotype Ontology (HPO) terms. The *p-value* of the chi-square test has to be under 0.05 to be considered statistically significant. MC, major criteria; mc, minor criteria; NA, non-applicable; SE, supportive evidence

	Clinical feature	HPO number	Frequency in the cohort	Frequency in the digenic triallelic cases [1]	Frequency in the monogenic biallelic cases [2]	Frequency in the "unknown" cases [3]	*p-value* [1-3]	*p-value* [1-2]
MC	Retinal dystrophy	HP:0000556	98% (78/80)	100% (24/24)	95% (18/19)	97% (36/37)	0.5442	0.2555
MC	Obesity	HP:0001513	90% (70/78)	91% (21/23)	84% (16/19)	92% (33/36)	0.6579	0.4798
MC	Postaxial polydactyly	HP:0100259	83% (66/80)	78% (18/23)	70% (14/20)	92% (34/37)	0.09483	0.5358
MC	Intellectual disability / Specific learning disability	HP:0001249 / HP:0001328	54% (38/71)	48% (11/23)	53% (8/15)	58% (19/33)	0.7718	0.74
MC	Abnormality of genital system	HP:0000078	48% (24/50)	44% (7/16)	56% (5/9)	48% (12/25)	0.8515	0.5706
MC	Abnormality of the kidney	HP:0000077	31% (15/49)	28% (5/18)	46% (5/11)	25% (5/20)	0.4711	0.3312
mc	Global developmental delay	HP:0001263	50% (27/54)	47% (7/15)	43% (6/14)	54% (14/25)	0.7003	0.8367
mc	Brachydactyly	HP:0001156	47% (15/32)	33% (5/15)	75% (3/4)	54% (7/13)	0.2686	0.1337
mc	Abnormal heart morphology	HP:0001627	25% (10/40)	21% (3/14)	22% (2/9)	29% (5/17)	0.857	0.9641
mc	Delayed speech and language development	HP:0000750	15% (4/27)	10% (1/10)	25% (3/12)	0% (0/5)	0.3607	0.3637
mc	Type II diabetes mellitus	HP:0005978	4% (3/78)	4% (1/24)	5% (1/20)	3% (1/34)	0.9259	0.8949
mc	Abnormality of the dentition	HP:0000164	3% (2/78)	0% (0/24)	0% (0/20)	6% (2/34)	0.265	NA
mc	Ataxia	HP:0001251	NA	NA	NA	NA	NA	NA
mc	Anosmia	HP:0000458	NA	NA	NA	NA	NA	NA
SE	Hearing abnormality	HP:0000364	8% (6/77)	4% (1/24)	5% (1/20)	12% (4/33)	0.4684	0.8949
SE	Aganglionic megacolon	HP:0002251	3% (2/78)	8% (2/24)	0% (0/20)	0% (0/34)	0.09934	0.1864
