# Supplementary material for: Allelic overload and its clinical modifier effect in Bardet-Biedl syndrome
Source: NPJ Genom Med. 2022 Jul 14;7:41. doi: 10.1038/s41525-022-00311-2 (PMC9283419; doi:10.1038/s41525-022-00311-2)
Supplement: Supplementary file 5 — Reporting Summary Checklist [file 41525_2022_311_MOESM5_ESM.pdf]

nature portfolio

Corresponding author(s): Diana Valverde and Carmen Ayuso

Last updated by author(s): May 3, 2022

Reporting Summary

Nature Portfolio wishes to improve the reproducibility of the work that we publish. This form provides structure for consistency and transparency in reporting. For further information on Nature Portfolio policies, see our Editorial Policies and the Editorial Policy Checklist.

Statistics

For all statistical analyses, confirm that the following items are present in the figure legend, table legend, main text, or Methods section.

n/a	Confirmed	
☐	☒	The exact sample size (n) for each experimental group/condition, given as a discrete number and unit of measurement
☐	☒	A statement on whether measurements were taken from distinct samples or whether the same sample was measured repeatedly
☐	☒	The statistical test(s) used AND whether they are one- or two-sided<br>*Only common tests should be described solely by name; describe more complex techniques in the Methods section.*
☒	☐	A description of all covariates tested
☒	☐	A description of any assumptions or corrections, such as tests of normality and adjustment for multiple comparisons
☒	☐	A full description of the statistical parameters including central tendency (e.g. means) or other basic estimates (e.g. regression coefficient) AND variation (e.g. standard deviation) or associated estimates of uncertainty (e.g. confidence intervals)
☒	☐	For null hypothesis testing, the test statistic (e.g. F, t, r) with confidence intervals, effect sizes, degrees of freedom and P value noted<br>*Give P values as exact values whenever suitable.*
☒	☐	For Bayesian analysis, information on the choice of priors and Markov chain Monte Carlo settings
☒	☐	For hierarchical and complex designs, identification of the appropriate level for tests and full reporting of outcomes
☒	☐	Estimates of effect sizes (e.g. Cohen's d, Pearson's r), indicating how they were calculated

Our web collection on statistics for biologists contains articles on many of the points above.

Software and code

Policy information about availability of computer code

Data collection	For oligogenic prediction, two different in silico tools were used (DiGePred and ORVAL), as described in Materials and Methods. Both are open source tools that can be found online in http://www.meilerlab.org/index.php/servers and https://orval.ibsquare.be/.
Data analysis	For molecular and oligogenic analysis/reanalysis, commercial clinical exome sequencing approaches and a custom in-house pipeline were used (https://github.com/TBLabFJD/Var; version v2.0.0), as described in previous works (see Materials and Methods).

For manuscripts utilizing custom algorithms or software that are central to the research but not yet described in published literature, software must be made available to editors and reviewers. We strongly encourage code deposition in a community repository (e.g. GitHub). See the Nature Portfolio guidelines for submitting code & software for further information.

Data

Policy information about availability of data

All manuscripts must include a data availability statement. This statement should provide the following information, where applicable:

- Accession codes, unique identifiers, or web links for publicly available datasets
- A description of any restrictions on data availability
- For clinical datasets or third party data, please ensure that the statement adheres to our policy

NGS data are available in public, open access repositories such as the European Genome-Phenome Archive (EGA; https://www.ebi.ac.uk/ega/home; EGAD00001007022 and EGAS00001006368) and the Collaborative Spanish Variant Server (CSVS; http://csvs.babelomics.org/) as aggregated data. The rest of the data are available upon reasonable request.

Field-specific reporting

Please select the one below that is the best fit for your research. If you are not sure, read the appropriate sections before making your selection.

☒ Life sciences ☐ Behavioural & social sciences ☐ Ecological, evolutionary & environmental sciences

For a reference copy of the document with all sections, see nature.com/documents/nr-reporting-summary-flat.pdf

Life sciences study design

All studies must disclose on these points even when the disclosure is negative.

Sample size	Sample-size calculation was not relevant to this study.
Data exclusions	No data were excluded from the analyses.
Replication	All findings are reproducible.
Randomization	Randomization was not relevant to this study.
Blinding	Blinding was not relevant to this study. Sample information must be known by investigators.

Reporting for specific materials, systems and methods

We require information from authors about some types of materials, experimental systems and methods used in many studies. Here, indicate whether each material, system or method listed is relevant to your study. If you are not sure if a list item applies to your research, read the appropriate section before selecting a response.

Materials & experimental systems

n/a	Involved in the study	
☒	☐	Antibodies
☒	☐	Eukaryotic cell lines
☒	☐	Palaeontology and archaeology
☒	☐	Animals and other organisms
☐	☒	Human research participants
☒	☐	Clinical data
☒	☐	Dual use research of concern

Methods

n/a	Involved in the study	
☒	☐	ChIP-seq
☒	☐	Flow cytometry
☒	☐	MRI-based neuroimaging

Human research participants

Policy information about studies involving human research participants

Population characteristics	No covariate was relevant to the analysis.
Recruitment	We selected retrospectively all families with a genetic diagnosis of Bardet-Biedl syndrome in at least one affected member from patient registries at the Genetics and Genomics Department of the HU-FJD and through collaborators, in the period from 1991 to February, 2022.
Ethics oversight	Research Ethics Committee of the Fundación Jiménez Díaz University Hospital and the Galician Ethical Committee for Clinical Research

Note that full information on the approval of the study protocol must also be provided in the manuscript.
